# Gut *Akkermansia muciniphila*, *Prevotellaceae*, and *Enterobacteriaceae* spp. as Possible Markers in Women-Related Nutritional and Clinical Trials: Familial Mediterranean Fever Disease

**DOI:** 10.1089/whr.2024.0076

**Published:** 2024-10-10

**Authors:** Astghik Pepoyan

**Affiliations:** ^1^ Food Safety and Biotechnology Department, Scientific Research Institute of Food Science and Biotechnology, Armenian National Agrarian University, Yerevan, Armenia.; ^2^ International Association for Human and Animals Health Improvement, Yerevan, Armenia.

**Keywords:** placebo, microbiome, FMF women, gut *Enterobacteriaceae*, gut *Prevotellaceae*, gut *Akkermansia muciniphila*

## Abstract

**Background:**

Studies have shown that the gut microbiota of healthy men and men with familial Mediterranean fever (FMF) disease respond differently to placebo. Given the fact that the composition of the gut microbiota is different in men and women, this study aimed to describe in detail the placebo response of the gut microbiota in healthy and FMF women.

**Materials and Methods:**

The bacterial response to placebo was fully evaluated on a previous PhyloChip™ DNA microarray-based assay (GEO Series; accession number GSE111835).

**Results:**

The change in the total number of operational taxonomic units in healthy women exposed to placebo is more than that of healthy men, in contrast to FMF people (704 vs. 140 and 409 vs. 7560, respectively [*p* < 0.05]). Gut Firmicutes diversities are more sensitive to placebo, whereas *Akkermansia muciniphila* remained unchanged after the placebo administration for both healthy and FMF people. *Gut Prevotellaceae* and *Enterobacteriaceae* diversities of healthy subjects and FMF women are also almost unchanged from placebo. Meanwhile, only 56.35% of gut *Enterobacteriaceae* diversities in FMF men were placebo resistant.

**Conclusion:**

The response to a placebo varies depending on a person’s gender and health status. Healthy and FMF women’s placebo study groups could be avoided by excluding placebo-sensitive 704 of 18,725 and 409 of 18,725 bacterial diversities, respectively. Because the placebo causes changes in all gut bacterial phyla in healthy and FMF women, and only the representatives of Enterobacteriaceae and Prevotellaceae families and *A. muciniphila* spp. are not affected by placebo, these bacteria can be considered as possible markers in women-related nutritional/clinical trials. Data on the response of the gut microbiota in healthy women to placebo might be used in studies of diseases other than FMF. The response of gut bacteria from different taxonomic affiliations to placebo may provide a basis for uncovering the role of these bacteria in the gut–brain axis.

## Introduction

Interest in the history of the placebo (Latin “placere” from the word “placere” to please) has increased in the last decade due to the modern pace of conducting placebo-controlled trials, as well as the development of alternative and esoteric medicine methods. Placebo-dependent nutritional and clinical studies, when the results of active treatment are compared with a “treatment” with empty capsules or capsules with an inert substance, face additional difficulties because of the need to provide the necessary numbers of volunteers in the placebo research groups, the associated financial costs, and even the interpretation of the study results.^1–5^ Moreover, sometimes volunteers in the control and placebo groups have to withdraw from the main drugs of the disease during such studies, causing various degrees of “anxiety,” especially to the patients. The placebo response of healthy men’s gut microbiota has detail been discussed by Tsaturyan and co-authors.^6^ Unfortunately, this study only included male subjects,^6^ which the authors attribute to the “nature” of familial Mediterranean fever (FMF) disease, where most carriers of the disease are men.^7–8^ FMF is a fairly common disease in Armenia and in general in the countries of the Mediterranean region. The disease is constantly and comprehensively studied due to its high prevalence and monogenicity (caused by MEFV gene mutations).^9–12^ Long-term use of colchicine, the main drug for FMF, can disrupt the microbiota balance in patients.^7,10,11^ To solve dysbiotic problems, special attention is paid to the investigations of probiotics, the main “regulators” of intestinal microbiota.

Currently, microbiome studies increasingly reveal the role of microbiomes in various processes of hosts.^13–15^ This applies to people,^16–20^ animals,^21–26^ and plants.^27–32^ Our previous investigations on FMF disease have shown the decisive role of gut microbiota in the disease diagnosis, course, and regulation processes.^11^ Depending on the gender of patients, changes were observed in gut *Escherichia* and *Helicobacter cinaedi* diversities.^33–34^ The studies on gut microbiota composition in male volunteers have also shown that the gut microbiome “responds” differently to placebo in healthy and FMF male subjects.^6^ Specifically, it was found that despite all members of the family *Enterobacteriaceae* in healthy men did not respond to a placebo, placebo influenced the numbers of the investigated operational taxonomic units (OTUs) of *Enterobacteriaceae* spp. in patients.^6^ *Akkermansia muciniphila* spp. was described as resistant to placebo species in male patients with FMF and healthy controls, whereas the gut *Faecalibacterium, Blautia,* and *Clostridium* spp. were characterized as placebo sensitive.^6^ In addition, the association of the levels of erythrocyte sedimentation rate, C-reactive protein, and rheumatoid factor with the patient’s gender was announced.^7^

This study aims to assess the response of gut microbiota to placebo in healthy and FMF women to identify the diversities of placebo-resistant bacteria, which in particular may technically help to simplify the design of placebo-dependent nutritional/clinical studies.

## Material and Methods

The study has approvals from the Ethics Committees at the Armenian National Agrarian University and the Higher Education and Science Committee of Armenia (10-15-21AG, 21.10.2021).

For the first time, the placebo response of the gut microbiota of 15 non-FMF and 15 FMF female volunteers was fully assessed based on the results of previously performed by us PhyloChip™DNA microarray-based partially randomized placebo trial (GEO Series; accession number GSE111835).^10,11^ Participants were screened according to age, ethnicity, and blood clinical, biochemical, immunological, and genetic tests. M694V/M694V carriers with FMF >7 years were selected. The composition of the gut microbiota during the attack-free period was evaluated. During the studies, as usual, patients took their 1 mg of colchicine during the day. All volunteers signed a written agreement that they would participate in trials, during which they would take nutritional supplements. All participants were asked to avoid milk and fatty and salty foods; they had not taken hormones/chemotherapeutic agents/antibiotics/probiotics since the month before the investigations. Pharmaceutical-grade empty hard gelatin capsules conforming to GMP, USP, and SP standards (Vitamax-E LLC, Yerevan, Armenia) were used as a placebo. Subjects in the placebo group did not know they were using empty capsules, but their doctors knew the participants in the placebo group. A comparative analysis of gut microbiota composition was performed for the same women aged 18–50 years before and after a twice-daily placebo capsule administration for one month, focusing on 18,725 bacterial OTUs as described in studies of Tsaturyan and coauthors.^10^

For the DNA isolation from the fecal samples frozen at −80°C, the commercially available kits: ZR Fecal DNA MiniPrep™ (Zymo Research Corp., Irvine, CA, USA) and Ultraclean® Fecal DNA Isolation (MoBio Laboratories Inc., Carlsbad, CA, USA) were used simultaneously to minimize DNA “losses”. The concentration and purity of extracted DNA were assessed spectrophotometrically (NanoDrop Microvolume Spectrophotometers, Thermo Fisher, USA).

The primers: 27f.jgi and 1492r.jgi (bacteria specific and bacteria/archaea specific, respectively) were used to perform microarrays and prepare 16S rRNA clone libraries.

For statistical analysis, the Excel Multibase 2015 add-in (NumericalDynamics, Tokyo, Japan) was applied, as well as the Mann–Whitney *U* test and paired *t-test* (Excel 2016). *p* < 0.05 was considered statistically significant.

## Results

### Placebo (inactive “treatment”)-altered gut bacterial diversities in non-FMF and FMF women

**FIG. 1.**
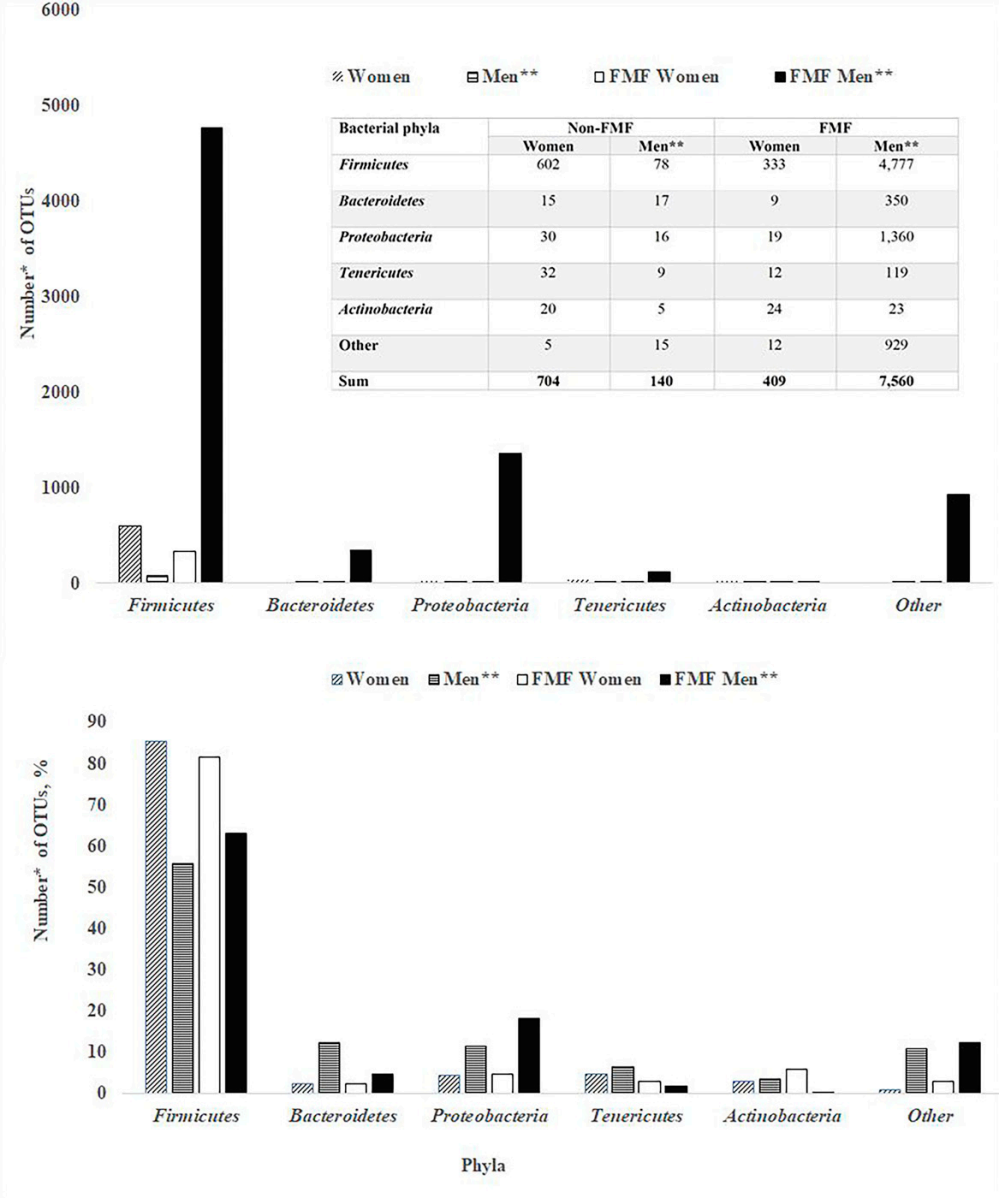
Comparative analysis of gut microbiota composition of healthy women and women with familial Mediterranean fever disease (FMF) before and after the placebo administration: The numbers of different operational taxonomic units (OTUs). *The impact of placebo on 18,725 bacterial OTUs was studied; *p* < 0.05. **Data were taken from a study on placebo-resistant gut bacteria in male patients with FMF.^6^

Figure 1 presents a comparative bacterial analysis of the gut microbiota at the phylum level in non-FMF and FMF women before and after placebo administration. According to Figure 1, the change in the total number of gut bacterial diversities in healthy women exposed to placebo is more than that of healthy men, in contrast to FMF people (704 vs. 140 and 409 vs. 7560, respectively [*p* < 0.05]). After placebo administration, there were changes in all bacterial phyla. In non-FMF women, placebo affected 602 OTUs of Firmicutes, 15 of Bacteroidetes, 30 of Proteobacteria, 20 of Actinobacteria, and 32 of Tenericutes (*p* < 0.05), whereas according to the investigations of Pepoyan and co-authors, in non-FMF men placebo affected 78 OTUs of Firmicutes, 17 OTUs of Bacteroidetes, 16 OTUs of Proteobacteria, 5 OTUs of Actinobacteria, and 39 OTUs of Tenericutes (*p* < 0.05) (Fig. 1). In contrast to healthy women, in women with FMF, the placebo affected 409 OTUs (*p* < 0.05): Firmicutes (333 OTUs), Bacteroidetes (9 OTUs), Proteobacteria (19 OTUs), Actinobacteria (24 OTUs), and Tenericutes (12 OTUs) (*p* < 0.05) (Fig. 1). Thus, a comparison of the results of the current studies with those of Pepoyan and colleagues showed that healthy women differed from healthy men as well as from women with FMF in the response of gut bacteria to placebo (Fig. 1). Of the 18,725 bacterial OTUs studied in healthy women, 3.76% were placebo-different bacterial OTUs, and in healthy men and women with FMF, the percentages were 0.75% and 2.18%, respectively. More sensitive to placebo were men with FMF (7560 OTUs) (Fig. 1).

**FIG. 2.**
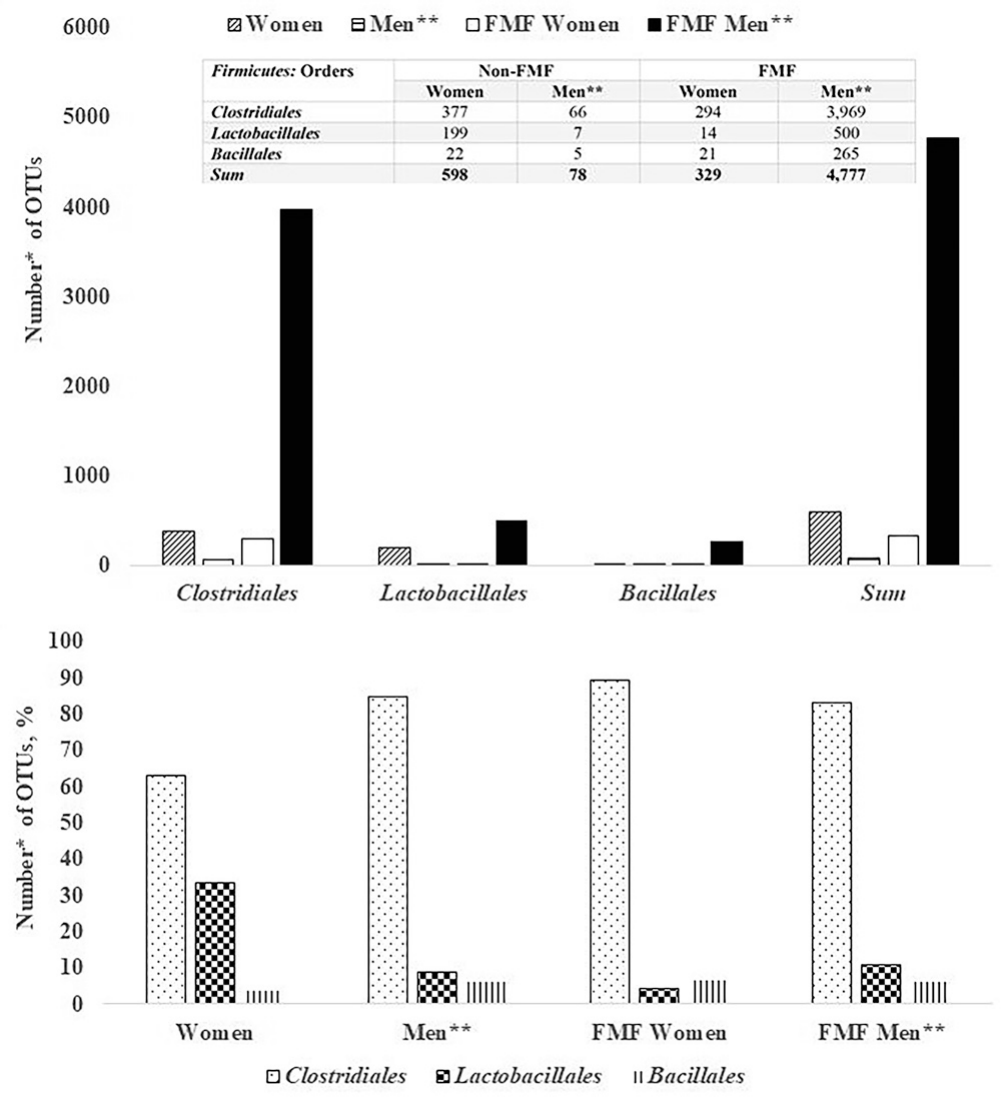
The numbers of placebo-altered gut Firmicutes diversities in healthy women and women with familial Mediterranean fever (FMF) disease. *The impact of placebo on 18,725 bacterial OTUs was studied; *p* < 0.05. **Data were taken from a study on placebo-resistant gut bacteria in male patients with FMF.^6^

Placebo-response investigations on the gut microbiota of healthy and FMF people show that from the Firmicutes the most sensitive to placebo bacteria are the Clostridiales (both for men and women) (Fig. 2). Placebo administration in healthy women has changed 598 OTUs, of which 377 belonged to Clostridiales, 199 to Lactobacillales, and 22 to Bacillales. Among FMF women, from the 329 OTUs, sensitive to placebo, 294 belonged to Clostridiales, 14 to Lactobacillales, and 21 to Bacillales. Of the 78 changed OTUs in healthy men, 66 belong to Clostridiales, 7 to Lactobacillales, and 5 to Bacillales (Fig. 2).

**FIG. 3.**
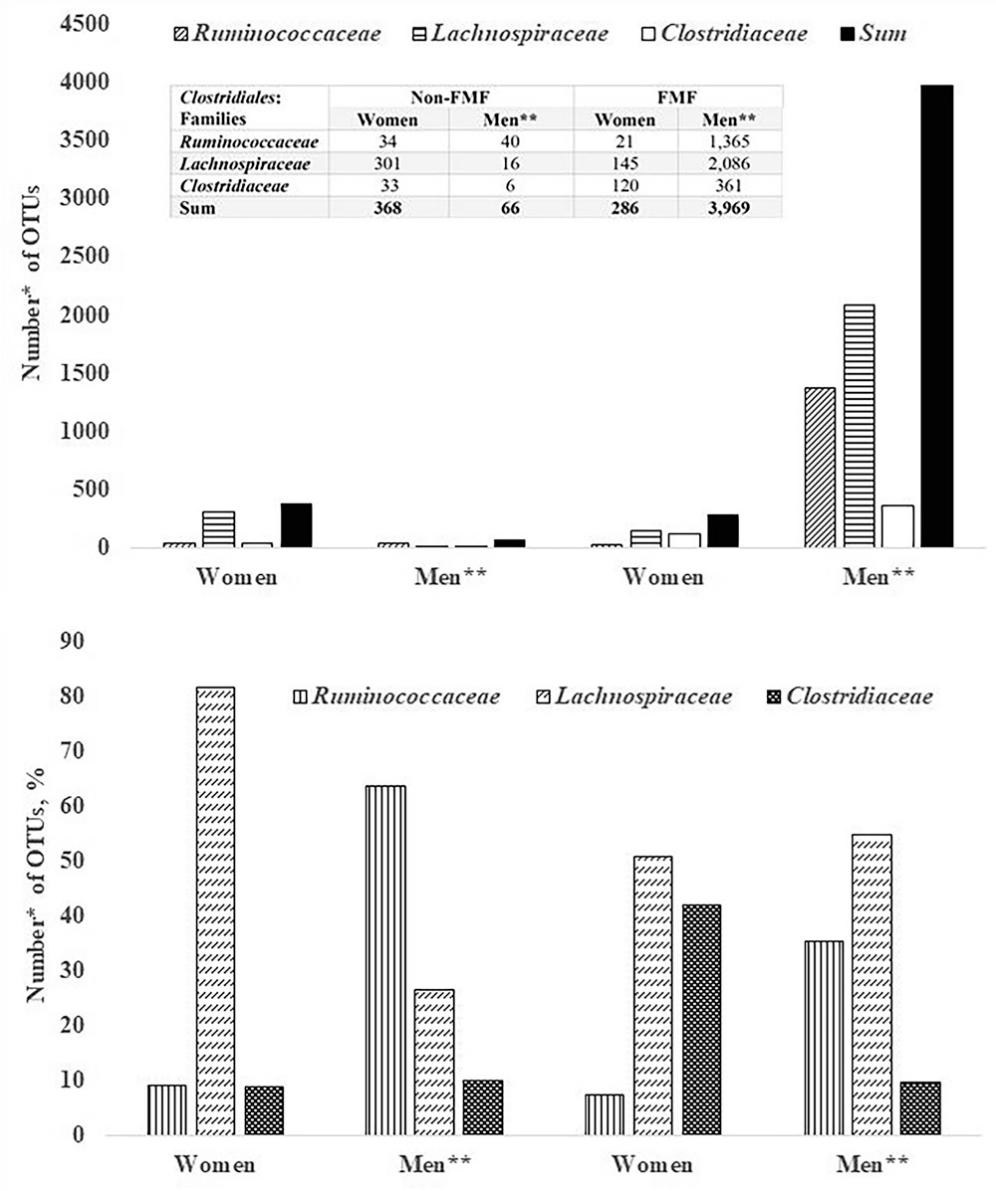
The numbers of placebo-altered gut Clostridiales diversities in healthy women and women with familial Mediterranean fever (FMF) disease. *The impact of placebo on 18,725 bacterial OTUs was studied; *p* < 0.05. **Data were taken from a study on placebo-resistant gut bacteria in male patients with FMF.^6^

Placebo-response investigations on the gut Clostridiales diversities in healthy women and women with FMF (Fig. 3) show that, unlike men, in women, placebo has the greatest effect on the family of Lachnospiraceae: the percentage of the altered number of OTUs of Lachnospiraceae diversities in healthy women was 81.79%, while in FMF women it was 41.96% (Fig. 3).

### Bacterial diversities sensitive to placebo in both healthy and FMF women

This study found that 49 of 18,725 bacterial OTUs examined were simultaneously susceptible in healthy and diseased women. 47 OTUs of them belong to the phylum Firmicutes.

### Placebo-resistant gut bacterial diversities in non-FMF and FMF women

*A. muciniphila* spp. remained unchanged after the placebo administration (Table 1). Of the 18,725 bacterial OTUs examined to reveal placebo-resistant bacterial diversities, 1228 belong to *Enterobacteriaceae*. Placebo mostly does not affect the number of OTUs of *Enterobacteriaceae* in FMF women, and in healthy women, only an OTU was affected after placebo administration (Table 1). Another important bacterial diversities in terms of the placebo response, represented by 747 OTUs, belong to the Prevotellaceae family. Placebo-resistant Prevotellaceae OTUs were 99.33% in healthy women and 100% in FMF women (Table 1).

**Table 1.** The Numbers of Placebo-Resistant Gut *Enterobacteriaceae*, *Prevotellaceae*, and *Akkermansia muciniphila* Diversities in Healthy Women and Women with Familial Mediterranean Fever (FMF) Disease

OTUs^*^	Non-FMF^***^		FMF^***^	
	Women	Men^**^	Women	Men^**^
*Enterobacteriaceae* spp.	1227 (99.92)	1226 (99.51)	1228 (100)	692 (56.35)
*Prevotellaceae* spp.	742 (99.33)	743 (99.46)	747 (100)	743 (99.46)
*A. muciniphila*	107 (100)	107 (100)	107 (100)	107 (100)

^*^ The impact of placebo on 18,725 bacterial operational taxonomic units (OTUs) was studied; *p* < 0.05.

^**^ Data were taken from a study on placebo-resistant gut bacteria in male patients with FMF.^6^

^***^ In parentheses—the percentages of OTUs.

## Discussion

The studies of placebo responses on physicochemical and physiological processes are of great importance both in nutritional/clinical investigations and in improving the effectiveness of therapeutic interventions through the use of treatment expectations.^35–38^ Such placebo-dependent studies also refer to probiotics.^38–39^ Probiotics, mainly bacteria characteristic of human^11,33,40^ and animal microbiota^41,42^ as well as plants,^43,44^ have regulatory effects on host organisms.^45–46^ Lactobacilli are the most popular probiotics (also, for one health probiotics).^44,47^ Having with the host organisms unique symbiotic, sometimes even parasitic relationships, they also provide the owner’s relationship with the environment.^40,44,48^ In addition to lactobacilli, probiotics from other origins are also widely discussed, e.g. *Faecalibacterium prausnitzii*, which is described as a next-generation probiotic (NGP).^49,50^ Another genera with probiotic characteristics are found in the mammals’ gut, *Prevotella* and *Blautia*, whose representatives are also widely discussed as NGPs.^50,51^ Some species of *Clostridium* and *Ruminococcus* are currently classified as *Blautia* spp.^50^ *Akkermansia muciniphila* probiotic strains are also evaluated as NGP.^51,52^ Interestingly, according to current studies, these probiotic strains may have different sensitivities to placebo. However, the most variable diversities of the gut microbiota of the study participants after taking a placebo were in the Firmicutes phylum (Fig. 1). In healthy women, 85.51% of OTUs belonging to Firmicutes differ and in patients 81.42%. The percentages of different OTUs do not vary greatly between FMF and non-FMF women after the placebo administration: for Firmicutes 85.51% versus 81.42%, Bacteroidetes 2.13% versus 2.2%*,* Proteobacteria 4.26% versus 4.65%, Tenericutes 4.55% versus 2.93%, and Actinobacteria 2.84% versus 5.87%, respectively (Fig. 1). The data in Figure 2 show that in healthy women, the number of altered by the placebo diversities belonging to the order Lactobacillales increases, probably due to a quantitative decrease in the affected diversities of Clostridiales, too (Fig. 2). In women, high sensitivity to placebo has the Lachnospiraceae; the percentage of altered OTUs in healthy women was 81.79%, and in patients was 41.96% (Fig. 3). It is noteworthy that *Faecalibacterium* spp., which belong to the Ruminococcaceae family and have probiotic representatives, are also placebo-sensitive. In the gut microbiota of healthy women/men, 7/7 OTUs of *Faecalibacterium*, respectively, were detected (not shown in Fig. 3).

Of the 18,725 bacterial OTUs examined, only 49 were simultaneously susceptible in both non-FMF and FMF females. Interestingly, in non-FMF and FMF women, 95.2% of these placebo-sensitive OTUs belonged to Firmicutes.

Data from this study indicate that the placebo causes changes in all gut bacterial families in healthy and FMF women, except for the Enterobacteriaceae and Prevotellaceae families. *A. muciniphila* spp. also remained unchanged under the influence of placebo. As for male patients with FMF, the placebo affects all gut bacterial families, except the family of Prevotellaceae (Table 1). In these patients, only 56.35% of *Enterobacteriaceae* remain unchanged.

The abovementioned results indicate that the current study poses new, still unexplained questions about placebo: What role do gender-independent placebo-sensitive gut bacterial communities play in the gut–brain axis in healthy and diseased individuals, and what role do gut bacterial communities that are sensitive or resistant to placebo, regardless of the person’s health status? Answers to these questions would help advance nutritional and clinical investigations on women and men.

Recently, the characterization of gut bacterial properties has also received attention in healthy-diseased gut microbiome comparisons.^53,54^ According to Tang and co-authors, compared with healthy subjects, the number of intestinal *E. coli* diversities is lower in patients with colorectal cancer than in healthy individuals, probably due to changes in the growth characteristics of *E. coli*.^53^ Gut bacterial growth characteristics might be important in evaluating not only the host’s health status^55^ but also in evaluating the effects of pre- and probiotics, as well as medicins’ effects, which require “placebo” comparisons, too.^54^ Bacteria other than *E. coli* (e.g., lactobacilli) that may be susceptible to placebo are also being actively investigated in dietary studies requiring placebo control groups. A healthy placebo study group could be avoided by excluding only 704 of 18,725 bacterial OTUs (Fig. 1) in a female nutritional/clinical trial. The complete list of 704 diversities will be discussed/published in future studies. These 704 OTUs do not include *A. muciniphila*, Prevotellaceae, and *Enterobacteriaceae* spp., which makes it possible to rely on changes in these bacteria at this time in nutritional/clinical studies without a placebo group of healthy women. According to FMF disease, if 409 diversities should be avoided in FMF women’s studies, the number is 7,560 for FMF men (Fig. 1).

### Limitations

Limitations of this study related to the limited study subjects, short-term changes in gut microbiota after placebo administration, disease, and colchicine intake by patients, and PhyloChip™DNA microarray limitations, requiring further studies.

## Conclusion

Placebo is an inactive substance/“treatment,” which is used mainly in clinical/nutritional studies as a control, taking into account the testee’s positive attitude toward the tested drug/nutrient. The response of gut microbiota to placebo depends on gender and healthcare status. The change in the total number of gut bacterial diversities in healthy women exposed to placebo is more than that of healthy men, in contrast to FMF people. Gut Firmicutes, the basis for the proposal of many probiotics, are more sensitive to placebo, whereas *A. muciniphila* diversities do not respond to placebo. Gut *Prevotellaceae* and *Enterobacteriaceae* diversities of healthy subjects and FMF women are also almost unchanged from placebo, which will make it possible to rely on the changes in these bacterial diversities in nutritional investigations for healthy women and particularly for FMF women in nutritional/clinical investigations.

Future studies will aim to elucidate the role of placebo-sensitive/resistant gut bacteria from different taxonomic affiliations in the gut–brain axis and probiotic therapy.

## Data Availability

Research data are available from the author.

## Abbreviations Used

*A. muciniphila*: *Akkermansia muciniphila*
FMF: familial Mediterranean fever
NGP: next-generation probiotic
OTU: operational taxonomic units
